# [Corrigendum] Apelin-13 attenuates ER stress-associated apoptosis induced by MPP^+^ in SH-SY5Y cells 

**DOI:** 10.3892/ijmm.2026.6026

**Published:** 2026-10-26

**Authors:** Yunlu Jiang, Haiqing Liu, Bingyuan Ji, Zhengwen Wang, Chunmei Wang, Chunqing Yang, Yanyou Pan, Jing Chen, Baohua Cheng, Bo Bai

Int J Mol Med 42: 1732-1740, 2018; DOI: 10.3892/ijmm.2018.3719

Following the publication of the above article, an interested reader drew the authors' attention to the fact that, regarding the light microscopic cellular images shown in Fig. 2A on p. 1735, the '100 *μ*M' and '500 *μ*M' (MPP^+^) data panels contained an overlapping section, such that data which were intended to show the results from differently performed experiments had apparently been derived from the same original source.

The authors were able to consult their original data, and realize that the data panel shown to represent the 100 *μ*M MPP^+^ experiment had inadvertently been copied across to show the 100 *μ*M MPP^+^ experiment. The revised version of Fig. 2, now showing the correct data for the '500 *μ*M' data panel in Fig. 2A, is shown on the next page. The authors confirm that the error they made in compiling this figure did not have a significant impact on either the results or the conclusions reported in this study, and all the authors agree with the publication of this Corrigendum. The authors are grateful to the Editor of *International Journal of Molecular Medicine* for allowing them the opportunity to publish this Corrigendum; furthermore, they apologize to the readership of the Journal for any inconvenience caused.

## Figures and Tables

**Figure 2.**
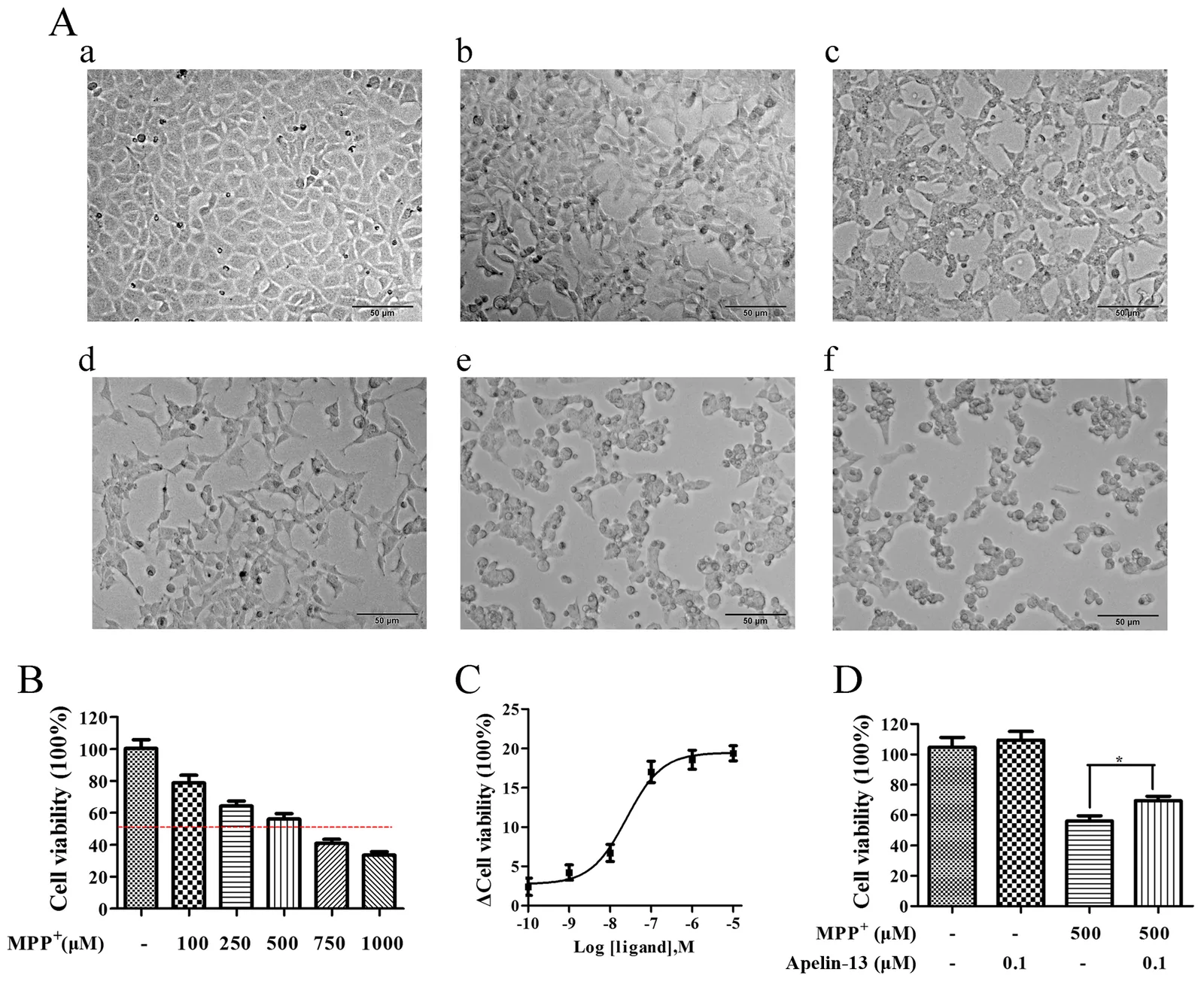
Apelin-13 protects SH-SY5Y cells against MPP+-induced damage. (A) Representative images from light microscopy analysis of SH-SY5Y cells treated with the indicated concentrations of MPP+ for 24 h. (B) Viability of SH-SY5Y cells treated with 0-1,000 *μ*M MPP+ for 24 h was assessed by the CCK-8 assay. (C) Dose-response curve of the effects of apelin-13 on the death of SH-SY5Y cells induced by treatment with 500 *μ*M MPP+. (D) Effects of apelin-13 on MPP+-induced SH-SY5Y cell death assessed by the CCK-8 assay. Values are presented as the mean ± standard deviation of three independent experiments. ^*^P<0.05 vs. MPP+-treated cells. MPP+, 1-methyl-4-phenylpyridine; CCK-8, Cell Counting Kit-8.

